# Supplementary material for: Inhibition of Nematocyst Discharge from Pelagia noctiluca (Cnidaria: Scyphozoa)—Prevention Measures against Jellyfish Stings
Source: Mar Drugs. 2022 Sep 8;20(9):571. doi: 10.3390/md20090571 (PMC9501295; doi:10.3390/md20090571)
Supplement: Supplementary file 1 [file marinedrugs-20-00571-s001.zip › marinedrugs-1876711-supplementary.pdf]

Supplementary Material

Table S1. ANOVA test for differences between formulation. Significance value: *** $p < 0.001$.

ANOVA					
**Df**	**Sum Sq**	**Mean Sq**	***F* value**	**Pr(>*F*)**	**Significance**
5	45201	9040	31.42	<2e-16	***
92	26467	288			

Table S2. Pair wise tests for differences between formulation. Significance values: *** $p < 0.001$.

**Formulations**	**10% Lidocaine in ethanol**	**1.5% Hydroxyacetophenone in distilled water + butylene glycol**	**3% Symsitive® in butylene glycol**	**Butylene glycol**
1.5% Hydroxyacetophenone in distilled water + butylene glycol	100.000	-	-	-
3% Symsitive® in butylene glycol	0.04754***	0.03893***	-	-
Butylene glycol	0.00159***	0.00110***	0.89229	-
Seawater	5.8×10^{-15}***	3.2×10^{-15}***	2.6×10^{-9}***	7.4×10^{-7}***
